# Intraperitoneal sertraline and fluvoxamine increase contextual fear conditioning but are without effect on overshadowing between cues

**DOI:** 10.1016/j.pbb.2014.12.004

**Published:** 2015-02

**Authors:** H.J. Cassaday, K.E. Thur

**Affiliations:** School of Psychology, University of Nottingham, Nottingham, NG7 2RD, UK

**Keywords:** Fear conditioning, Overshadowing, Contextual conditioning, 5-Hydroxytyptamine, Rat

## Abstract

Treatment with selective serotonin reuptake inhibitors (SSRIs) can reduce contextual conditioning. Since contexts comprise a variety of potentially competing cues, impaired overshadowing may provide an account of such effects. The present study therefore compared the effects of two SSRIs on overshadowing and contextual conditioning, testing suppression of an ongoing behavioral response (licking) by cues previously paired with foot shock. Conditioning to a 5 s light stimulus was reduced when it was presented in compound with a 5 s noise, thus overshadowing was demonstrated. In two experiments, this overshadowing was unaffected by treatment with either sertraline or fluvoxamine. However, unconditioned suppression to the noise (tested in a control group previously conditioned to the light alone) was reduced after sertraline (10 mg/kg, i.p.). The successful demonstration of overshadowing required the use of a second conditioning session or an additional conditioning trial within the same conditioning session. Neither weak nor strong overshadowing (of the light by the tone) was affected by any drug treatment. Moreover, counter to prediction, conditioning to contextual cues was increased rather than impaired by treatment with sertraline (10 mg/kg, i.p.) and fluvoxamine (30 mg/kg, i.p.).

## Highlights

• Setraline (10 mg/kg) increased latency to drink in the conditioning context.
• Fluvoxamine (30 mg/kg) reduced licking in the conditioning context.
• Discrete cue conditioning to an overshadowed light cue was unaffected.
• Setraline (10 mg/kg) reduced unconditioned suppression to the overshadowing noise.

## 1 Introduction

Contextual fear conditioning is typically reduced by both acute and chronic treatment with selective serotonergic reuptake inhibitors (SSRIs; Hashimito et al., 1996, Hashimito et al., 2009, Inoue et al., 2011, Li et al., 2006b, Montezinho et al., 2010, Nishikawa et al., 2007). Contexts comprise a configuration of potential conditioning cues which could compete with each other. Thus the above findings may reflect the role of serotonin (5-hydroxytryptamine, 5-HT) in fear conditioning per se; in other words, fear responses to competing cues might also be reduced by SSRIs.

In an overshadowing procedure, the relative intensity of competing discrete CSs modulates their capacity to become associated with an outcome (unconditioned stimulus, UCS). Normally a more intense CS acquires associative strength at the expense of a less intense CS (Pavlov, 1927). The neural substrates of overshadowing have been little investigated (Horsley et al., 2008, Nelson et al., 2011). If overshadowing is reduced, one possible outcome is learning failure, because no single cue acquires sufficient associative strength. Thus, if salience modulation is impaired, this could reduce overshadowing, and potentially reduce conditioning to contextual cues. The substrates of overshadowing are therefore of interest in relation to contextual conditioning.

In the present study, overshadowing was examined using a fear conditioning procedure that we have previously used to test the effects of amphetamine (Nelson et al., 2011). After pairing of a CS (or two CSs in compound) with footshock UCS, fear was later measured as the suppression of licking in water deprived rats, upon presentation of the CS(s). The same conditioned suppression procedure adopted to assess overshadowing was also used to determine the suppression of licking produced by the contextual cues provided by the conditioning box (Cassaday et al., 2001, Horsley and Cassaday, 2007, Norman and Cassaday, 2003). The effects of two SSRIs were examined in this fear conditioning procedure: sertraline (at 10 and 20 mg/kg i.p.; experiment 1) and fluvoxamine (at 15 and 30 mg/kg i.p.; experiment 2). Based on the fact that contextual conditioning impairment has been widely reported after treatment with SSRIs (Inoue et al., 2011), it was predicted that the overshadowing effect should also be impaired by these treatments.

## 2 Experimental procedures

### 2.1 Animals

For each experiment, 72 naïve adult male Wistar rats (Charles River, UK) were caged in pairs on a 12:12 h light/dark cycle with food and water *ad libitum*. They were handled for approximately 5 min per day for 1 week and then placed on water deprivation 24 h prior to the start of each experiment. The mean start weight was 215 g (range 192–237 g in experiment 1 and 200–238 g in experiment 2). The data from one rat were lost due to a procedural error (in experiment 1). The work was conducted in accordance with the UK Animals Scientific Procedures Act 1986, Project Licence: PPL 40/3163.

### 2.2 Drug treatments

Sertraline HCl (Tocris, UK) was dissolved in 2% Tween80 saline for administration of doses of 10 or 20 mg/kg (calculated as the free base) at 2 ml/kg injection volume. Fluvoxamine maleate (Tocris, UK) was dissolved in saline for administration of doses of 15 or 30 mg/kg (calculated as the free base) at 1 ml/kg injection volume (i.p.; warmed to dissolve at 30 mg/ml). Sertraline injections were made 30 min, and fluvoxamine injections were made 60 min prior to the conditioning stage of the procedure. Control rats were injected with the equivalent volume of vehicle.

### 2.3 Behavioral apparatus

Six identical fully automated conditioning boxes were housed within sound-attenuating cases containing ventilation fans (Cambridge Cognition, Cambridge, UK). The conditioning boxes were steel (25 cm × 25 cm × 22 cm high) with a Plexiglas door (27 cm × 21 cm high), inset at the front. A waterspout was mounted on one wall, 5 cm above the floor and connected to a lickometer supplied by a pump. Licks were registered by a break in the photo beam within the spout, which also triggered water delivery of 0.05 ml per lick. The waterspout was illuminated when water was available. Three wall-mounted stimulus lights and the house light were set to flash on (0.5 s) and off (0.5 s) for a total 5 s duration. In both experiments, these flashing lights served as the CS for the control rats. In the overshadowing group, the 5 s flashing lights CS was presented in compound with a 5 s mixed frequency noise set at 85 dB, delivered by a loudspeaker set in the roof. Footshock of 1 s duration and 1 mA intensity provided the UCS. This was delivered via the grid floor (steel bars 1 cm apart) by a constant current shock generator (pulsed voltage: output square wave 10 ms on, 80 ms off, 370 V peak under no load conditions; MISAC Systems, Newbury, UK). Stimulus control and data collection were by an Acorn Archimedes RISC computer programmed in Basic with additional interfacing using an Arachnid extension (Cambridge Cognition).

### 2.4 Behavioral conditioning procedures

Water deprivation was introduced 1 day prior to shaping. The rats then had 1 hr and 15 min of *ad libitum* access to water in their home cage after each of the procedural stages described below. This home cage access was in addition to any water drunk in the conditioning boxes (available from the apparatus waterspout on all days of the procedure apart from conditioning). Thus animals were trained, conditioned and tested, after 22 hours of water deprivation, on consecutive days.

#### 2.4.1 Pre-conditioning to establish baseline lick responses

In order to initiate licking behavior, rats were first placed in the conditioning boxes in pairs (with their cage mates) and were shaped for 1 day until all drank from the waterspout. No data were recorded. Thereafter, animals were individually assigned to a conditioning box for the duration of the experiment (counterbalanced by experimental group). There then followed 5 days of pre-training, in which rats drank in their conditioning boxes for 15 min each day (timed from first lick). The licking spout was illuminated throughout, but no other stimuli were presented. Latency to first lick was recorded to assess any pre-existing differences in readiness to drink.

#### 2.4.2 Conditioning with footshock

No water was available within the box, and the waterspout was not illuminated. In experiment 1, the UCS footshock was delivered following termination of the CS in each of 2 conditioning trials per conditioning session (of which there were 2). The first pairing of CS and UCS was presented after 5 min had elapsed, and the second pairing was 5 min after the first, followed by a further 5 min left in the apparatus. In the absence of licking, there were no behavioral measures to record. In experiment 2, three conditioning trials were delivered, as before 5 min apart within a 20 min single conditioning session. In both experiments, the CS was provided by the flashing lights, compounded with the noise stimulus in the overshadowing groups.

#### 2.4.3 Reshaping after footshock

On the day following conditioning, animals were reshaped, following the same procedure as in the pre-conditioning sessions. This both re-established licking after conditioning and provided a measure of contextual conditioning, reflected in the extent to which licking was suppressed in the conditioning boxes.

#### 2.4.4 Overshadowing tests

On the day following reshaping, the animals were placed in the conditioning boxes and presented with the CS. Water was available throughout the test, and the waterspout was illuminated. Once the animals had made 50 licks, the CS was presented for 15 min. The latency to make 50 licks in the absence of the CS (the A period, timed from the first lick made in each box) provided a measure of any individual variation in baseline licking. This was compared with the time taken to complete 50 licks following CS onset (B period) in a suppression ratio (A/(A + B)) to assess the level of conditioning to the CS, adjusted for any individual variation in drink rate. In experiment 1 only, rats underwent a second conditioning session. Following completion of the above procedure, an additional baseline day as per pre-conditioning, was used to re-establish licking. There then followed the same behavioral procedure as before.

### 2.5 Experimental design and analysis

In both experiments, there were 6 experimental groups run in a 2 × 3 independent factorial design (n = 11–12/cell): conditioning group at levels control or overshadowing; drug at levels saline, 10 or 20 mg/kg sertraline, and saline, 15 or 30 mg/kg fluvoxamine, in experiments 1 and 2 respectively. The same design was applied to analyses of variance (ANOVAs) for the pre-conditioning baselines (to check for pre-existing differences by experimental condition-to-be), the reshaping latencies and number of licks made within the first 5 min (to measure differences in contextual conditioning), suppression to the CS and suppression to the competing tone stimulus (to measure discrete cue conditioning). Post hocs to further examine significant effects of drug were by Tukey test. In each case alpha was set at p < 0.05 for the rejection of the null hypothesis. The dependent variables were lick latencies and number of licks within the first 5 min at pre-conditioning and reshaping, and the A period and suppression ratio for the conditioning tests. Where necessary, raw latency data (time to first lick at reshape) were log transformed so that their distribution was suitable for parametric analysis. Non-significant effects on baseline lick responding are not reported.

## 3 Results

### 3.1 Experiment 1: effects of sertraline in an overshadowing procedure

#### 3.1.1 Reshaping: contextual conditioning

**Fig. 1:**
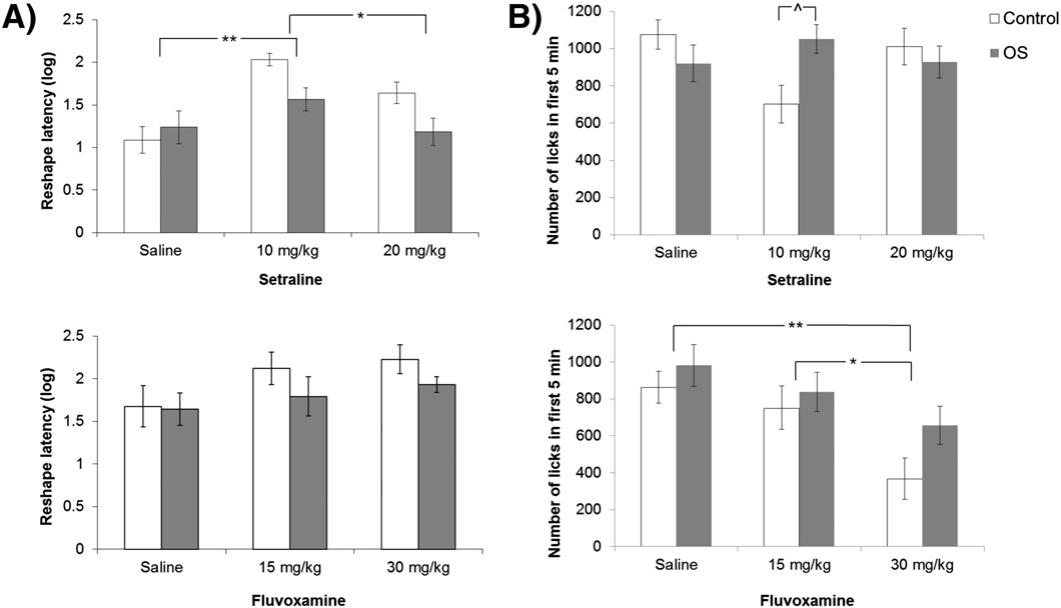
Contextual conditioning: effects of sertraline (10 and 20 mg/kg; experiment 1) and fluvoxamine (15 and 30 mg/kg; experiment 2) on suppression of licking in the conditioning boxes after 2 (experiment 1) or 3 (experiment 2) conditioning trials. Suppression was measured as (A) the (log) latency (s) to make the first licks in the first reshaping session and (B) the total number of licks made over the first five min of the 15 min reshaping session. Bars show the results by drug and conditioning group, in that the footshocks had either followed the light alone (control; white bars) or the light compounded with a noise in the overshadowing groups (OS; grey bars). Error bars show two standard errors of the mean for approximate between groups comparisons. Comparisons show post hoc tests of the effects of drug ^p < 0.06; *p < 0.05; **p < 0.001 by Tukey test.

Fig. 1A shows that following the first conditioning session, there were differences in latency to drink. Statistically, ANOVA showed both a main effect of conditioning group [*F*(1,66) = 4.784, p = 0.032] and drug [*F*(2,66) = 9.696, p < 0.001]. The main effect of drug arose because the 10 mg/kg sertraline group took overall longer to recommence licking than the saline group [p < 0.001; this difference in latency was not significant at 20 mg/kg, but the drug groups were different from each other, p = 0.027; Fig. 1]. There was a significant conditioning group by drug interaction on the overall number of licks made within the first 5 min of reshape [*F*(2,66) = 4.536, p = 0.014; Fig. 1B]. In the groups previously conditioned under 10 mg/kg sertraline, control conditioned rats tended to drink less than those previously conditioned with the compound CS [p = 0.052].

#### 3.1.2 Overshadowing tests

**Fig. 2:**
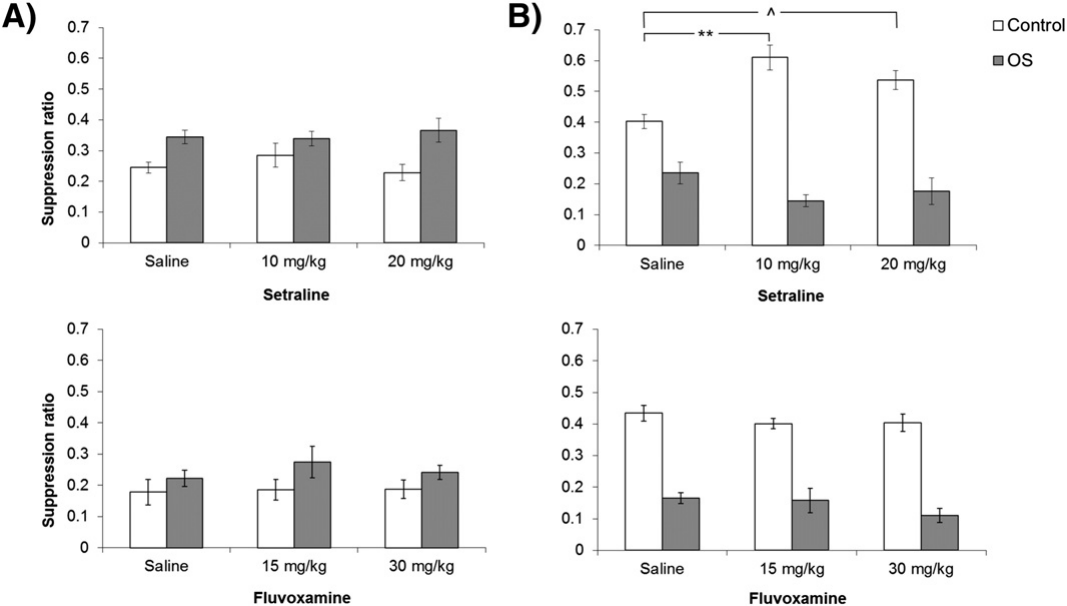
Overshadowing tests: effects of sertraline (10 and 20 mg/kg; experiment 1) and fluvoxamine (15 and 30 mg/kg; experiment 2) after in total 4 (experiment 1) or 3 (experiment 2) conditioning trials. Conditioned suppression was measured after conditioning to (A) the flashing light stimulus and (B) the noise stimulus. The level of conditioning is expressed as mean suppression ratio (calculated as A/(A + B); where A was the time taken to complete 50 licks prior to any stimulus presentation and B was the time taken to complete 50 licks during stimulus presentation). Bars show the results by drug and conditioning group, in that the footshocks had either followed the light alone (control; white bars) or the light compounded with a noise in the overshadowing groups (OS; grey bars). Error bars show two standard errors of the mean for approximate between group comparisons. Comparisons show post hoc tests of the effects of drug ^p < 0.06; *p < 0.05; **p = 0.001 by Tukey test.

After the first conditioning session, there was no significant effect of conditioning group on suppression to the light CS [*F*(1,66) = 0.300] neither was there any effect of drug [maximum *F*(2,66) = 0.681]. The second conditioning session produced a robust overshadowing effect [*F*(1,65) = 17.277, p < 0.001]. Fig. 2A shows that control rats' licking was more suppressed when the light was presented, compared to licking in the corresponding overshadowed group. However, there was no effect of drug, either overall or in interaction with conditioning group [maximum *F*(2,65) = 1.059]. On the noise test, there was again a main effect of conditioning group [*F*(1,65) = 151.409, p < 0.001]. As expected, the compound conditioned groups suppressed licking during the noise presentation, whereas there was little unconditioned suppression to the noise in the groups which had been conditioned with the light alone. There was no main effect of drug [*F*(2,65) = 1.621], but there was a drug by conditioning group interaction [*F*(2,65) = 10.563, p < 0.001]. Fig. 2B shows that the 10 mg/kg sertraline group subsequently showed less unconditioned suppression to the noise (tested in the control group previously conditioned to the light alone) than their saline-treated counterparts [p = 0.001; this difference was marginal at 20 mg/kg, p = 0.053].

### 3.2 Experiment 2: effects of fluvoxamine in an overshadowing procedure

#### 3.2.1 Reshaping: contextual conditioning

There was no overall difference in the time taken to start licking at reshape; the effect of drug did not reach significance [*F*(2,66) = 2.503, p = 0.090]. However, there was a significant main effect of drug on the overall number of licks made in the first 5 min [*F*(2,66) = 7.501, p = 0.001; Fig. 1B]. Post hoc comparisons confirmed that rats treated with 30 mg/kg fluvoxamine drank overall less than the saline-treated [p = 0.001]; as well as less than those in the 15 mg/kg drug condition [p = 0.031]. This pattern is consistent with the non-significantly longer latencies shown in Fig. 1A for comparison.

#### 3.2.2 Overshadowing tests

On the light test suppression ratio measure, there was a main effect of conditioning group [*F*(1,66) = 4.717, p = 0.033]. Fig. 2A shows that there was overall less suppression in the overshadowed groups. There were no significant effects of drug, either overall or in interaction with conditioning group [maximum *F*(2,66) = 0.357]. ANOVA of the noise test suppression ratios shown in Fig. 2B confirmed a significant effect of conditioning group [*F*(1,66) = 164.888, p < 0.001], in the absence of any effect of drug or interaction [maximum *F*(1,66) = 1.408].

## 4 Discussion

Contexts confront the organism with a variety of potentially competing cues. If the normal ability to apportion associative strength on the basis of the relative salience of contemporaneously presented cues is impaired, then one possible outcome is learning failure. For example, contexts lacking in distinctive features, such as a salient background odor or noise, might fail to engage the attentional processes necessary for successful associative learning. Thus, reduced contextual conditioning could in principle result from reduced overshadowing. In the present study, both sertraline (experiment 1) and fluvoxamine (experiment 2) increased contextual conditioning, albeit with some differences in the profile of action and in relation to dose. However, despite these clear effects on contextual conditioning, and the difference in subsequent responding to the overshadowing noise stimulus (seen after conditioning under sertraline in experiment 1), neither treatment had any effect on overshadowing.

### 4.1 Contextual conditioning measured at reshaping

Increased contextual conditioning manifested as longer latencies to drink, or a reduced number of licks in the first 5 min of the reshaping session. In experiment 1, rats previously treated with 10 mg/kg sertraline took longer to recommence licking than either the saline or the 20 mg/kg sertraline group. Moreover, there were some group differences, in that control conditioned rats treated with 10 mg/kg sertraline tended to drink less than those previously conditioned with the compound CS. These effects on contextual conditioning were seen only during the first reshaping session, in other words after 2 but not 4 conditioning trials, consistent with the possibility that (the drug effect on) the level of contextual conditioning was related to the development of overshadowing. In experiment 2, rats previously conditioned under fluvoxamine (30 mg/kg) drank less at reshaping than both the saline- and 15 mg/kg-treated rats. These lower levels of licking are again consistent with increased contextual conditioning after drug treatment. Drug effects did not differ by conditioning group, and the reshape latencies were not greater in rats conditioned under fluvoxamine. As discussed below, there was a procedural difference between experiments 1 and 2 in that three conditioning trials were used (delivered within a single session). This change was made in order to optimize the overshadowing aspect of the procedure.

### 4.2 Overshadowing and suppression to the competing cue

The overshadowing procedure was identical to that used previously (Nelson et al., 2011). However, in the present study, overshadowing took more than two conditioning trials to emerge. Test suppression to the light was not different in control and compound conditioned groups after two conditioning trials. Accordingly, in order that drug effects on overshadowing could be examined, a second conditioning session was administered in experiment 1, and in experiment 2 three conditioning trials were delivered within a single conditioning session. However, when overshadowing emerged (after a total of 4 conditioning trials in experiment 1, and 3 trials in experiment 2) there was no effect of acute treatment with either sertraline or fluvoxamine, at either of the doses tested. The reduced expression of unconditioned suppression to the noise stimulus shown in the sertraline behavioral control groups must have been indirectly mediated, because the noise was not present at conditioning (when the drug treatments were administered) for the behavioral control groups. In both experiments, the noise component of the compound cue was nonetheless effective in accruing associative strength. Intact overshadowing suggests that ‘bottom-up’ salience gating, based on the relative salience of competing cues, is impervious to attenuation by the treatments examined here.

### 4.3 Why should SSRIs affect contextual but not discrete cue conditioning?

Although treatments with SSRIs generally impair contextual conditioning, they also typically increase discrete cue conditioning in standard single cue procedures (Inoue et al., 2011). Earlier evidence also suggested that serotonergic effects on discrete cue and contextual conditioning are dissociable, in this case after 5-HT depletion produced by 5,7-dihydroxytryptamine injected into the cerebral ventricles (Cassaday et al., 2001). Moreover, the same dissociation is well-documented in studies of conventional lesions to the hippocampus (Hirsh, 1974, Phillips and Le Doux, 1992, Selden et al., 1991, Winocur et al., 1987), and micro-injection studies have shown that hippocampus mediates the reduced contextual conditioning produced by 5-HT_1a_ agonists in freezing studies (Li et al., 2006a).

Overshadowing is a cue competition effect normally seen between two discrete cues presented in a compound. Since contexts present multiply compounded cues, cue competition of the kind measured in discrete cue overshadowing procedures could provide a mechanism relevant to understanding the dissociation between the effects of SSRIs on contextual and discrete cue conditioning. Good contextual conditioning might rely, for example, on a salient cue such as a distinctive odor or background sound ‘standing out’ sufficiently to capture attention. In the absence of any particularly attention grabbing cue, contexts might be expected to support generally lower levels of contextual conditioning. However, the results of the present study lend no support to the hypothesis that overshadowing determines the level of contextual conditioning supported. The present results show that discrete cue overshadowing was unaffected at the same time that contextual conditioning was increased. Moreover, although it is very well documented that contextual conditioning is—for whatever reason—generally weaker than that supported by discrete CSs, the results of the present study do not support the view that apparently dissociable effects on discrete cue and contextual conditioning are attributable to a difference in the baseline level of conditioning. Specifically, the lack of drug effects on overshadowing was shown at variable levels of suppression, after different numbers of conditioning trials.

### 4.4 Methodological differences from previous studies

The vast majority of earlier studies have examined contextual conditioned freezing rather than discrete cue conditioning procedures (Inoue et al., 2011). Acute (and chronic) treatment with SSRIs is typically found to reduce the acquisition and retrieval of contextual conditioned fear assessed in freezing procedures (Inoue et al., 2011). The acquisition-mediated effect may, however, be less reliable than that seen when SSRIs are administered before the retrieval test, as is more typical (Hashimito et al., 1996, Inoue et al., 2004, Li et al., 2006a, Li et al., 2006b). In the present study, the drug treatments administered acutely at the conditioning stage of the procedure were unlikely to have much direct effect in the subsequent test stages, as the half life of SSRIs in the rat is much shorter than the 24 hour separation between the stages of the experimental procedure (Tremaine et al., 1989). Moreover, in line with the present findings, others have reported that acute treatment with SRRIs increased contextual conditioning when administered before acquisition, while decreasing the expression of contextual fear conditioning when applied before the retrieval test (Gravius et al., 2006, Montezinho et al., 2010). Thus, apparently inconsistent results may be related to methodological differences between studies. What can be firmly concluded from the present study is that contextual conditioning was modulated by SSRI treatments which were without effect on overshadowing measured under directly comparable conditions in the same rats.

### 4.5 How was increased contextual conditioning mediated?

Although a useful first step to increase intrasynaptic 5-HT levels (Kitaichi et al., 2010), SSRIs are not actually selective to the serotonergic system and in fact modify the function of all the monoamines in vivo (Stanford, 1996, Hughes and Stanford, 1996). Additionally, with respect to 5-HT they are indirect agonists and thus nonspecific. Synergistic effects of 5-HT_1a_ agonists and SSRIs are consistent with 5-HT_1a_ mediation of effects on emotionality (Zanoveli et al., 2007). Similarly, co-administration of SSRIs and tandospiron (a 5HT_1a_ receptor agonist) inhibited contextual freezing after fear conditioning (Nishikawa et al., 2007). Therefore, in the present study, contextual conditioning could in principle have been modulated by an action at the 5-HT_1a_ receptor sub-type. However, it must be noted that conditioning to contextual cues was increased rather than inhibited after SSRI treatment in the present study, and a role for other receptor subtypes is not excluded.

### 4.6 Conclusions

In the present study, treatment with SSRIs increased rather than impaired conditioning to contextual cues. The same treatments had no effect on the competition between discrete cues which results in overshadowing. Thus, contrary to expectation, there was no evidence to suggest that effects on overshadowing may explain the observed differences in contextual conditioning seen after treatment with sertraline or fluvoxamine. Rather, the observed pattern of results lends further support to the contention that contextual and discrete cue conditioning depend on different neuropharmacological substrates.
